# A Case of Painless Excision

**DOI:** 10.5005/jp-journals-10005-1499

**Published:** 2018-04-01

**Authors:** Ipshita A Suyash, Rupinder Bhatia

**Affiliations:** 1 Postgraduate Student, Department of Pediatric and Preventive Dentistry, D Y Patil School of Dentistry, Navi Mumbai, Maharashtra, India; 2 Professor, Department of Pediatric and Preventive Dentistry, D Y Patil School of Dentistry, Navi Mumbai, Maharashtra, India

**Keywords:** Laser, Painless dentistry, Peripheral giant cell granuloma, Soft tissue lesions.

## Abstract

Soft tissue lesions of the oral cavity are seen in children at the dental office. This case report aims to showcase the ability of laser to treat recurrent soft tissue lesions in the oral cavity in a painless manner. This painless procedure provides relief to the child and parent who suffer from anxiety toward dental treatment.

**How to cite this article:** Suyash IA, Bhatia R. A Case of Painless Excision. Int J Clin Pediatr Dent 2018;11(2):135-140.

## BACKGROUND

Believed to be an idiopathic non-neoplastic proliferative lesion, peripheral giant cell granuloma (PGCG) or giant cell epulis has been previously stated to be a reparative granuloma.^1^ However, the incongruity of its progression overruled its reparative nature. Though debatable, when it comes to etiological factors and symptoms, it is destructive if not treated. Theories suggest it to be a reactive, inflammatory, or even an endocrine pathology.^2^ Earlier, it was considered as a true tumor, given its vast destructive capabilities. Other synonyms are giant cell epulis, giant cell reparative granuloma, osteoclastoma, or giant cell hyperplasia.^3^

Peripheral giant cell granuloma is usually found in adults with highest prevalence rate in 4th and 6th decade.^4^ It is not encountered in children on a daily basis but has been reported in them. Giansanti and Waldron^5^ noted the incidence rate of 20 to 30% in 1st and 2nd decades of life. Shafer et al^6^ and Giansanti and Waldron^5^ implied that PGCG generally occurs in the incisor and canine region; however, Pindborg^7^ confirms the common site of occurrence to be the molar and premolar region.

Out of 12 cases described in the scientific journals, 5 patients were aged less and 5 more than 10 years of age, girls are commonly affected, and PGCG is often located on the gingiva as well as on the alveolar mucosa in the posterior region of the maxilla. In the present case, a lesion was found in maxillary right posterior region.

Peripheral giant cell granuloma is relatively aggressive in its progression in children. The factors assigning it an aggressiveness title are its size, its extension to neighboring tissues, and its ability to relapse, associated bone resorption, permanent teeth displacement, and induced mobility of primary teeth surrounding the lesion.^8^

Clinically, PGCG is a smooth brown, red, or bluish nodule, sessile or pedunculated, with a slight predilection for the posterior segments of the jaws.^4,9^ These lesions vary from a few millimeters to 4 cm in diameter. Case reports state this lesion to occur 2 times more commonly in females than in male subjects and there is a frequent predilection for the mandible than the maxilla.

Etiological factor causing PGCG, however, stays indefinite. Constant local irritation by either faulty restorations or dental prosthesis, extraction sites where root stumps are left behind, plaque, calculus, and food accumulation are considered to lead to its development.^10^ Trauma and irritation after an orthodontic management is also a causative factor for the apparition of a PGCG.^11^ Levine et al^12^ and Grand et al^13^ have described the association of a dental trauma and PGCG, wherein the lesion was seen to have occurred within 6 months posttrauma.

There is a high rate of relapses which is seen to occur with respect to PGCG and to limit both irreversible bony destruction and extraction of permanent teeth. Some authors advocate radical and extensive excision of PGCG which comprises not only the excision of the gingival lesion, but also of the adjacent periosteum and sometimes the superficial bony layer. The current case is also a case report treating the relapsed PGCG.^14^

Aggressive treatments are avoided in children, considering the ongoing growth. Laser excision was carried forth and the results were clinically significant.

## CASE REPORT

**Fig. 1:**
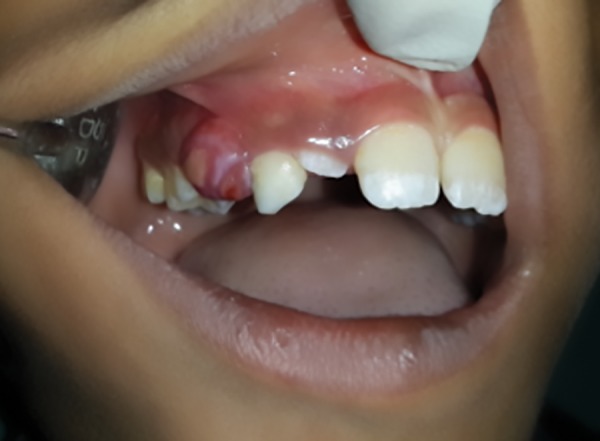
Preoperative view in 8-year-old male patient showing intraoral swelling

**Fig. 2:**
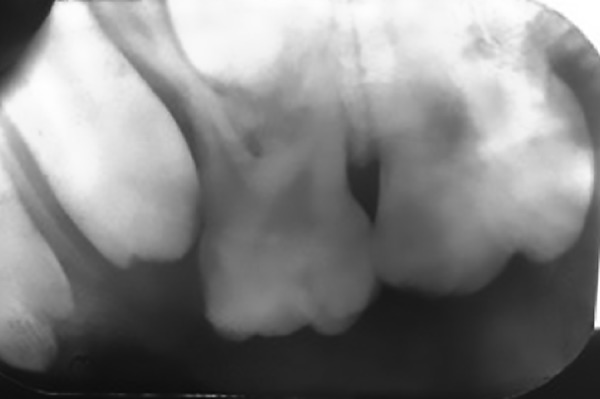
Preoperative radiograph of lesion

**Fig. 3:**
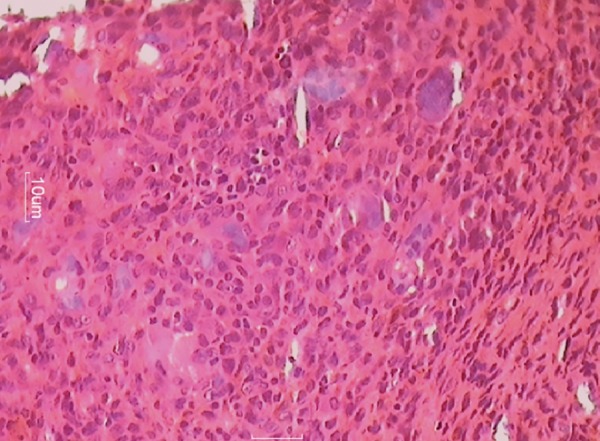
Local anesthesia administration

**Fig. 4:**
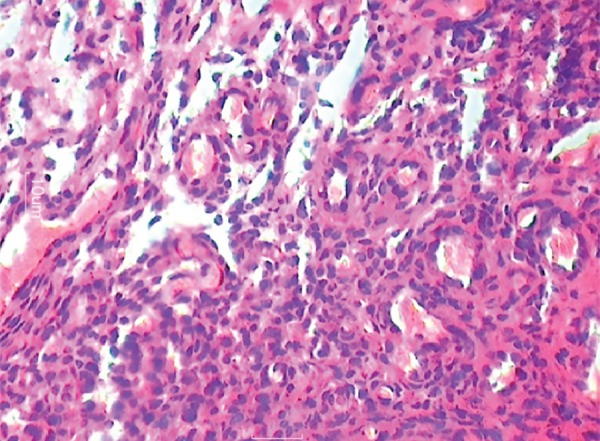
Laser excision

**Fig. 5:**
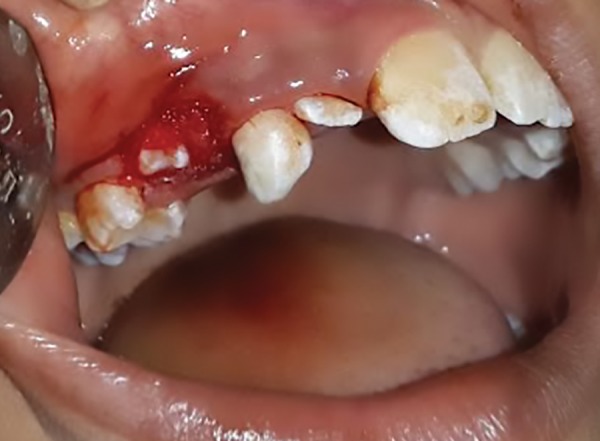
Postlaser excision

**Fig. 6:**
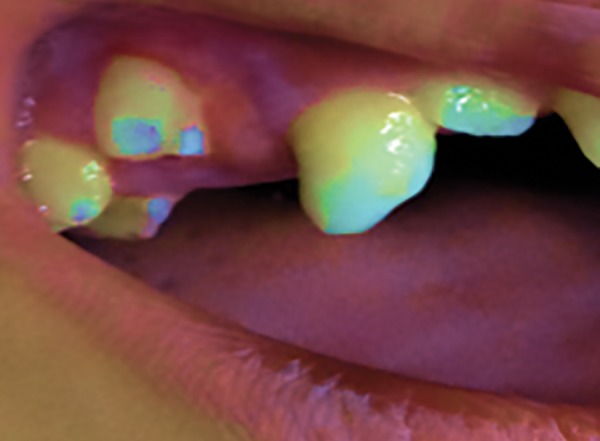
Postlaser excision (24-hour late follow-up)

An 8-year-old boy came to the Department of Pediat-ric and Preventive Dentistry in Dr. D Y Patil School of Dentistry, Navi Mumbai, India, with the chief complaint of a boil in the upper right back region enlarging since 6 months. Complete medical and dental history of the parents and the child was taken. The parents disclosed a similar lesion to have occurred 6 months ago in the same region, which had been excised with a scalpel by a general dentist (Figs 1 to 6).

No sutures were given, allowing it to heal by secondary intention. No other relevant medical history surfaced. On clinical examination, the “boil” was a sessile lesion of 1.5 × 0.5 × 1 cm in dimension. It exhibited a reddish hue, was fluctuant, and bled on slight examination with finger. There was no blanching or exudate seen.

**Fig. 7:**
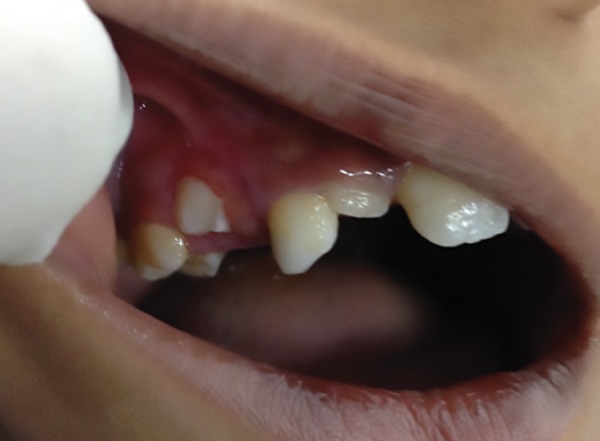
Six months follow-up

**Fig. 8:**
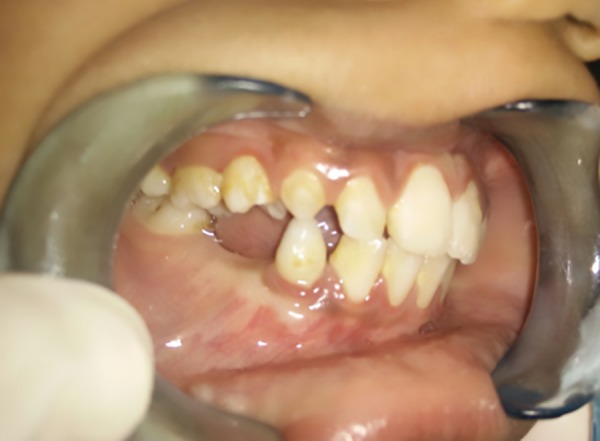
Twenty-four months follow-up

**Fig. 9:**
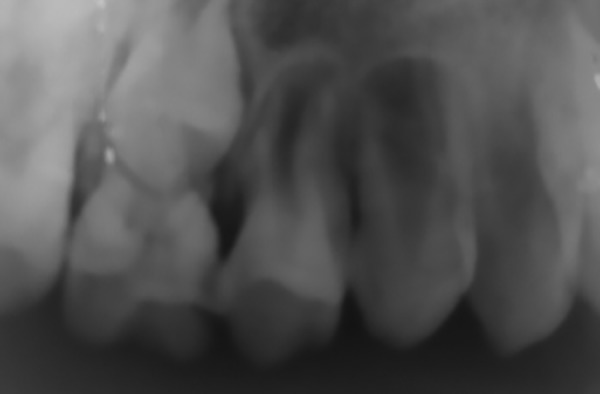
Postoperative 20 months follow-up radiograph

**Fig. 10:**
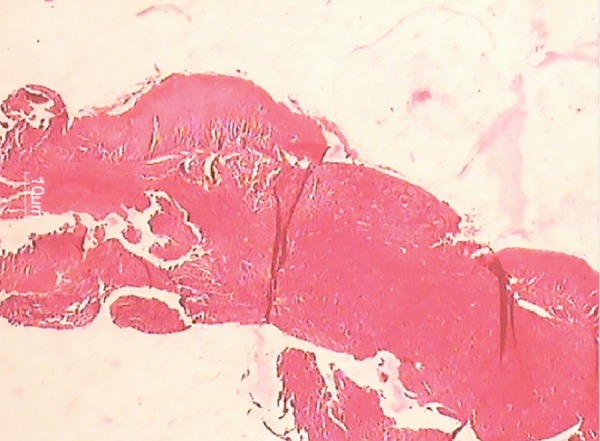
Histopathological report

**Fig. 11:**
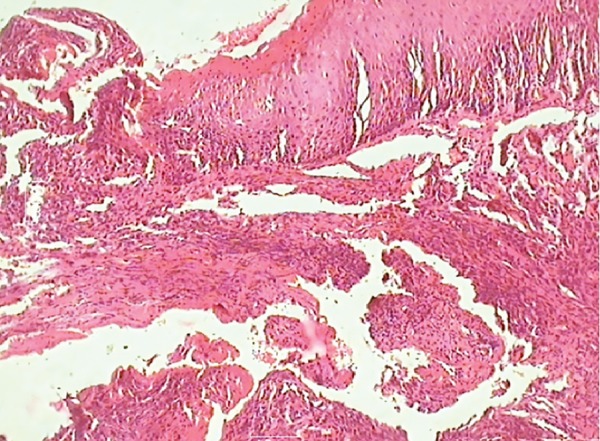
Giant cells visible on low magnification

Intraoral periapical radiograph showed a radiolu-cency surrounding the developing premolar. There was also constant trauma being inflicted to this area due to grossly carious lower right molars, which impinged the area. Extraction was considered for the same to eradicate the underlying irritant.

**Fig. 12:**
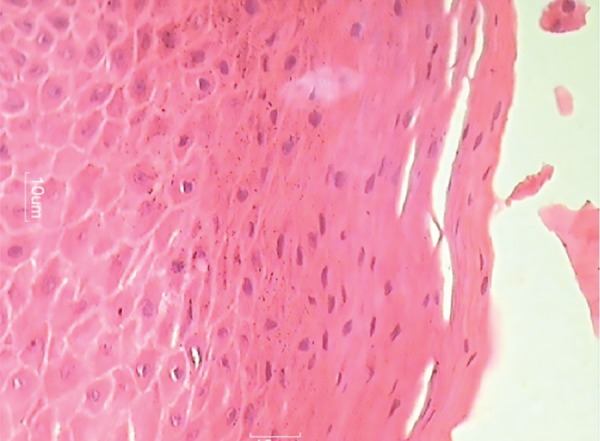
Giant cells seen at the periphery

**Fig. 13:**
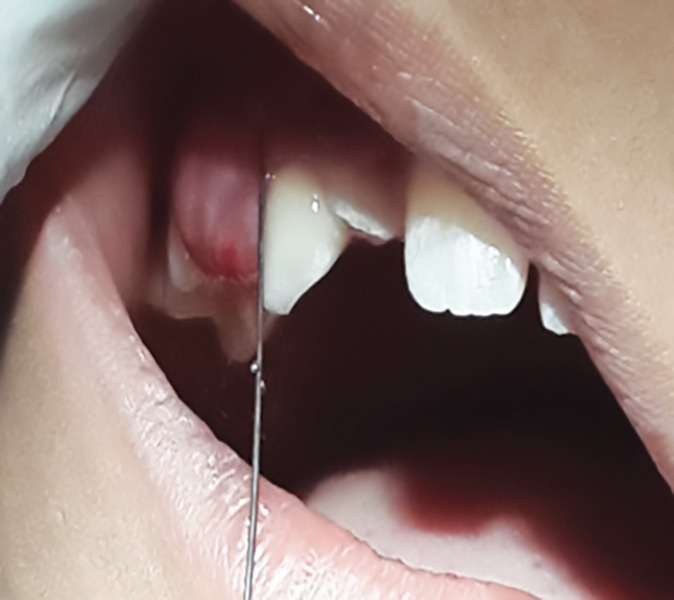
Giant cells seen

**Fig. 14:**
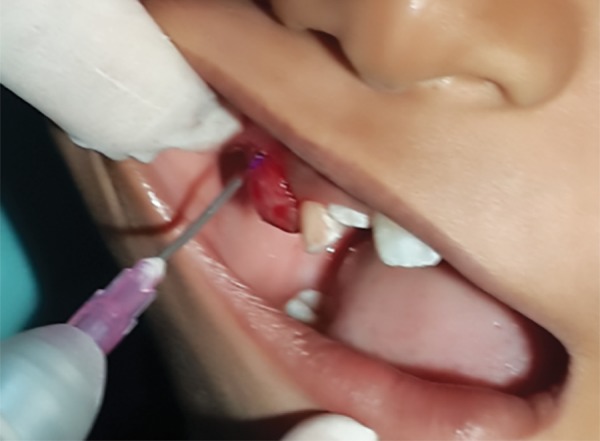
Giant cells (high magnification)

The differential diagnosis for the same lesion was pyogenic granuloma, PGCG, peripheral ossifying fibroma, inflammatory fibrous hyperplasia, and peripheral odontogenic fibroma. Excision with a soft tissue diode laser was carried forth. Local anesthesia was administered to ensure minimal bleeding in the region and reduce any discomfort for the child (Figs 7 to 14).

The child’s behavior rating was of Frankel rating 3 (positive). The excision was uneventful. The gingival mass was excised and sent for histopathological consideration. Vitamin E in the form of Evian oil-based capsule was topically applied. The patient’s parents were asked to apply it for the following 3 days twice daily. The patient was recalled the next day and then the next week.

The 7-day follow-up revealed the presence of the premolar erupting and gingiva to be coral pink and unharmed. The excised lesion was analyzed under hematoxylin and eosin stain. Histological report described nodular tumor in the subepithelium separated by fibrous tissue.

The report stated there to be frequent multinucleated giant cells in stroma containing ovoid to spindle-shaped cells. The stroma was elaborately vascularized and contained rare inflammatory cells, such as lymphocytes, plasma cells, and eosinophils along with hemosiderin at the tumor periphery. Bony tissue included was histologically unremarkable. The histological features confirmed it as PGCG.

Postoperative healing was uneventful. The patient was followed up at 1, 3, 6, 12, 15, and 18 months. The premolar surrounded by the excised lesion is seen to erupt as per physiological process.

## DISCUSSION

This lesion accounts for <10% of all hyperplastic gingi-val lesions.^15^ The theory states the capacity of PGCGs to enlarge to be 0.1 to 3 cm and 94% of such lesions are <1.5 cm.^16^ The extent of these lesions rarely crosses 2 cm in diameter, although larger ones may be seen occasionally.^17^ Their gradual growth, however, becomes a tumorous mass, which then counters normal oral function.^18^ Inconsistent growth patterns exhibited by these lesions dissuade one to measure their expansion capacity. In the present case, the size of the lesion was 1.5 cm.

Unique in size, the lesion in question required special care during the excision. Known to either be sessile or pedunculated, PGCG spread by penetrating through the periodontal membrane. They cause a break in the continuity of the membrane by opening externally or internally (ulcerative lesions) in the region they occur in.^18^

Pathologically, they mimic various other lesions. For example, the pyogenic granuloma is difficult to discriminate from a PGCG based on clinical features alone. A pyogenic granuloma is also a soft friable nodule, which bleeds spontaneously. However, radiographic differences exhibited by PGCG of displacing teeth and resorbing the surrounding alveolar bone differentiates it from a pyogenic granuloma.

Another soft, friable swelling of the gingiva is the parulis. Etiologically seen to develop due to a trapped local irritant, gingival pocket and/or nonvital teeth, a purulent exudate seen associated with it distinguishes this inflammatory lesion from a PGCG.

Hemangiomas are red and/or blue-hued congenital lesions. These vascular malformations increase in size with age, spontaneously bleed, are warm to touch, and blanch when palpated. These are easily differentiated from PGCG.

The peripheral ossifying fibroma, a reactive gingival growth, shares similar clinical features as the PGCG. However, it lacks the purplish blue hue associated with a PGCG and the calcifications seen in its radiographs differentiate the two lesions from one another.

Radiographic characters are generally nonsuggestive, but in a PGCG, the aggressive destruction around the alveolar margin or crest of bone when teeth are associated with the granuloma makes it an important diagnostic medium for these lesions. For this reason, the destructive central giant cell granuloma that appears within the jaw itself is comparatively distinguished from a PGCG by radiographic diagnosis.

In the present case report, an intraoral periapical radiograph demonstrated focal loss of the alveolar crestal bone in deciduous first maxillary molar region.

Due to the large size of the lesion, an excisional laser biopsy and histopathologic evaluation were done for the diagnosis of the progressively enlarging gingival mass. Medical history was thoroughly taken to exclude hyper-parathyroidism, and tests were considered to evaluate serum calcium, phosphate, alkaline phosphatase, and parathyroid hormone.^20^

Laser excision was considered over surgical scalpel excision to ensure painless treatment and removal of the aggressive lesion.

Diode lasers are effective tools for precise cutting^21^ and make minimal change to adjacent tissues. The laser vaporization method coagulates and seals small vessels providing no postoperative bleeding.^22^

Children experience less pain with diode laser. This is because the thermal necrosis created by the laser through vaporization of the tissue seals sensory nerves, decreasing their ability to transmit stimuli (of pain)^23,24^ and denaturation protein aids in decreasing pain.^25^ Diode laser proves to have not only a bactericidal effect but also an anti-inflammatory effect in the oral cavity, reducing chances of infection.^26^

## CONCLUSION

Laser treatment for aggressive lesions is considered more effective and efficacious. This painless treatment can be useful in children who suffer from the anxiety of surgical treatment and fear the sharp scalpel.^27,28^

## CLINICAL SIGNIFICANCE

In pediatric patients, identifying any lesion at its inception provides a possibility for a conservative approach. It helps deterring the long-term developmental flaws. Using lasers proves to be more efficient and is slowly replacing the old scalpel technique. Peripheral giant cell granuloma can show rapid growth and increase in size within a few months. Arising from the endothelial cells of the capillaries, periosteum, periodontal ligament, or connective tissue of the gingiva^8^ can disrupt the underlying bone, interfere with eruption of teeth, and may produce minor tooth movement.^29^ Radiographs play an essential role in confirming its origination from either the mucosa or periosteum and whether it penetrates the underlying bone damaging unerupted tooth.
